# Supplementary material for: Reduction in Lens Epithelial Cell Senescence Burden through Dasatinib Plus Quercetin or Rapamycin Alleviates D-Galactose-Induced Cataract Progression
Source: J Funct Biomater. 2022 Dec 21;14(1):6. doi: 10.3390/jfb14010006 (PMC9862066; doi:10.3390/jfb14010006)
Supplement: Supplementary file 1 [file jfb-14-00006-s001.zip › jfb-2056318-supplementary.pdf]

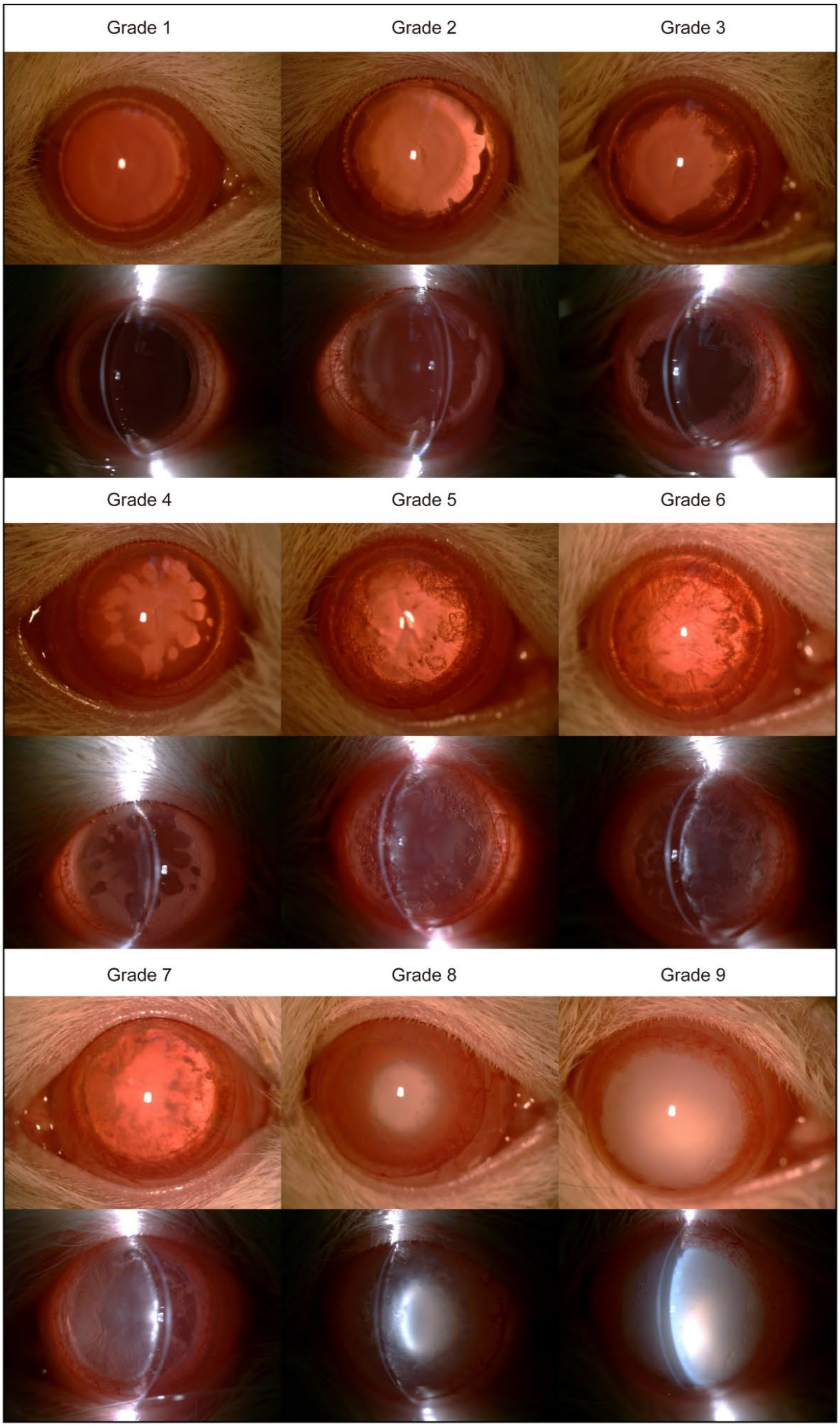

Figure S1. Opacity index for D-galactose induced cataract.

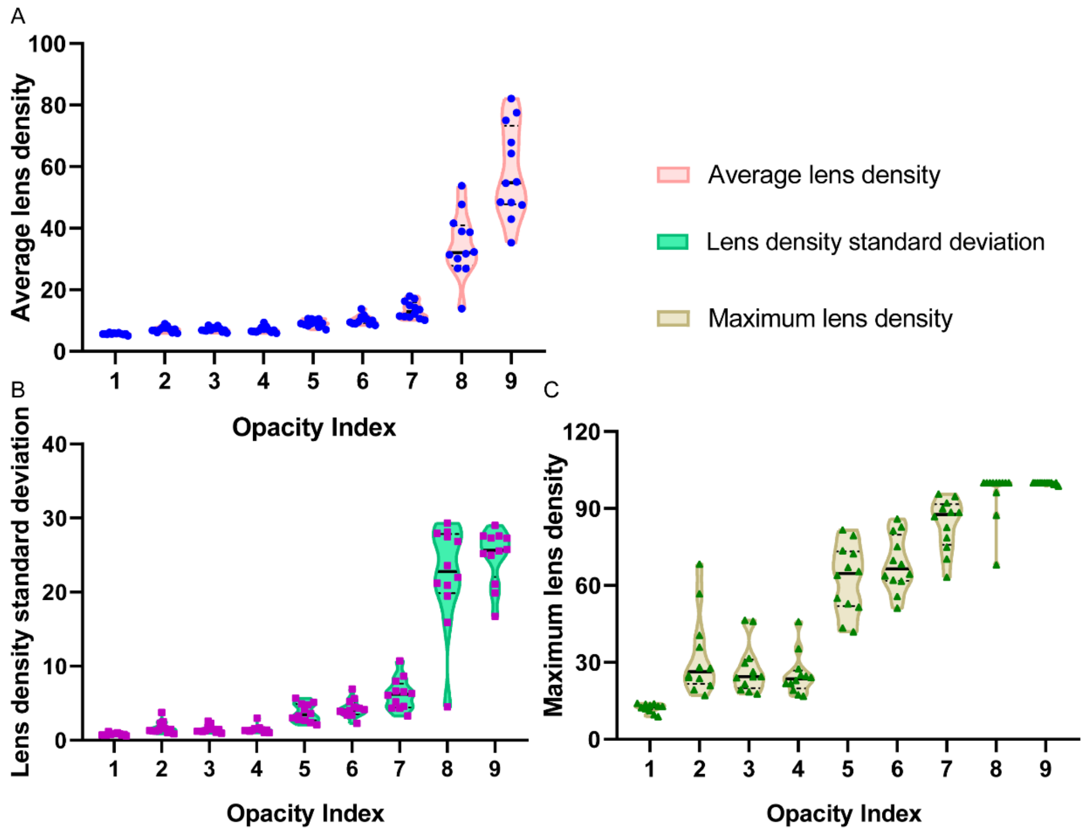

Figure S2. The association between opacity index and lens densities. A, the association between opacity index and average lens density. B, the association between opacity index and lens density standard deviation. C, the association between opacity index and maximum lens density.

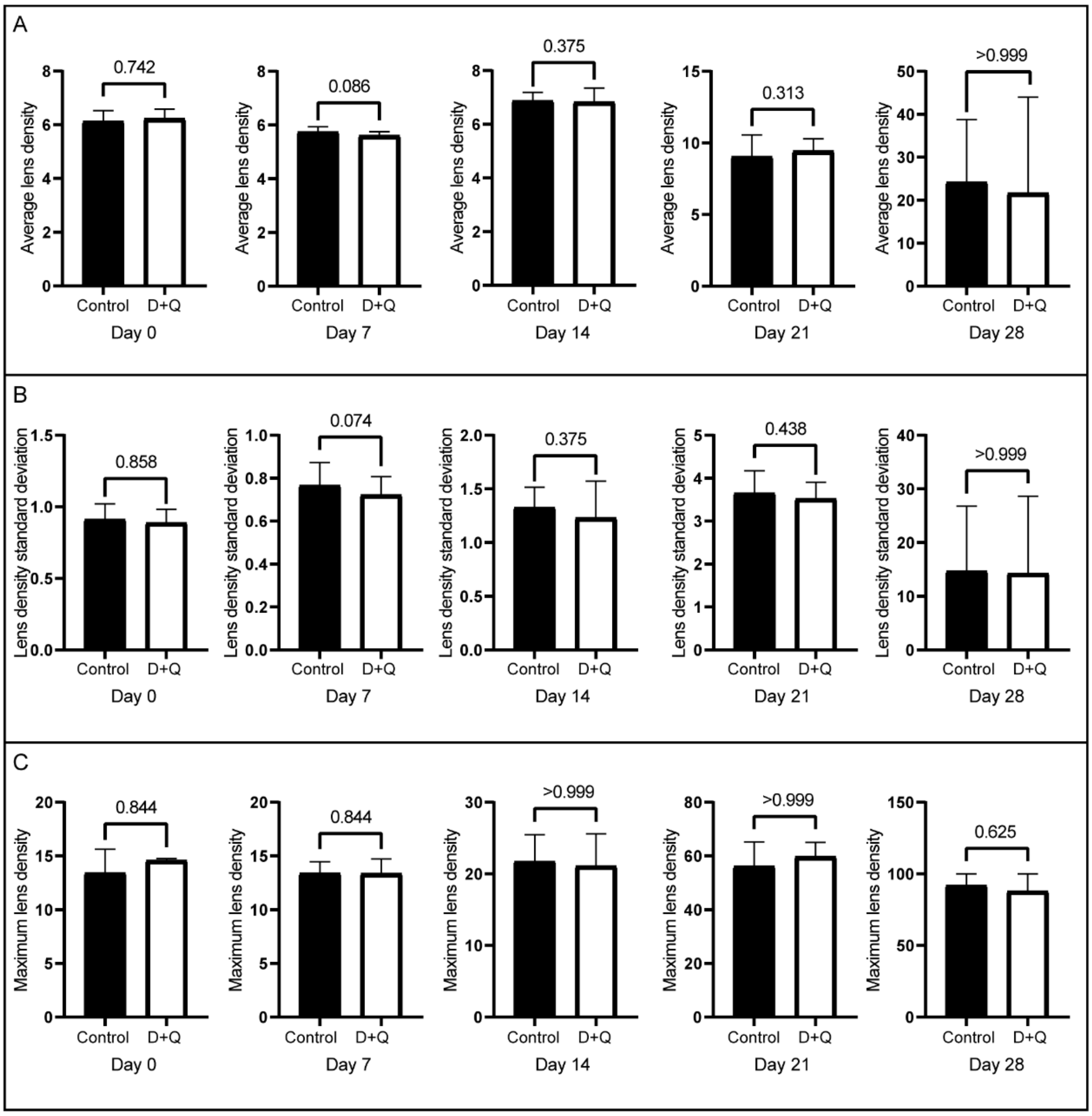

Figure S3. The comparisons between paired eyes from each cataract rats after administration of D+Q with low concentration. A, the comparison of average lens density between paired eyes. B, the comparison of lens density standard deviation between paired eyes. C, the comparison of maximum lens density between paired eyes. D, dasatinib; Q, quercetin.

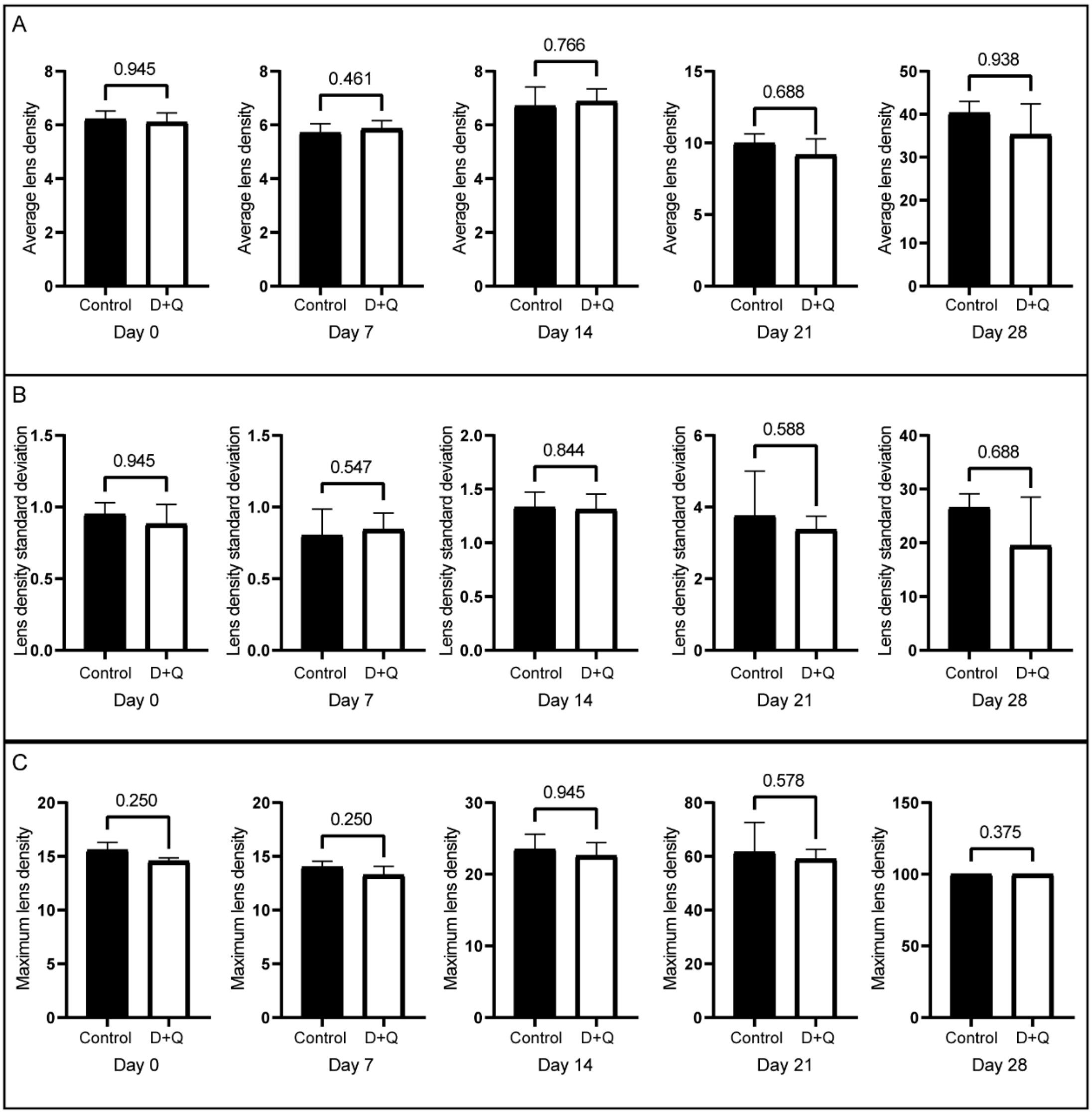

Figure S4. The comparisons between paired eyes from each cataract rats after administration of D+Q with moderate concentration. A, the comparison of average lens density between paired eyes. B, the comparison of lens density standard deviation between paired eyes. C, the comparison of maximum lens density between paired eyes. D, dasatinib; Q, quercetin.

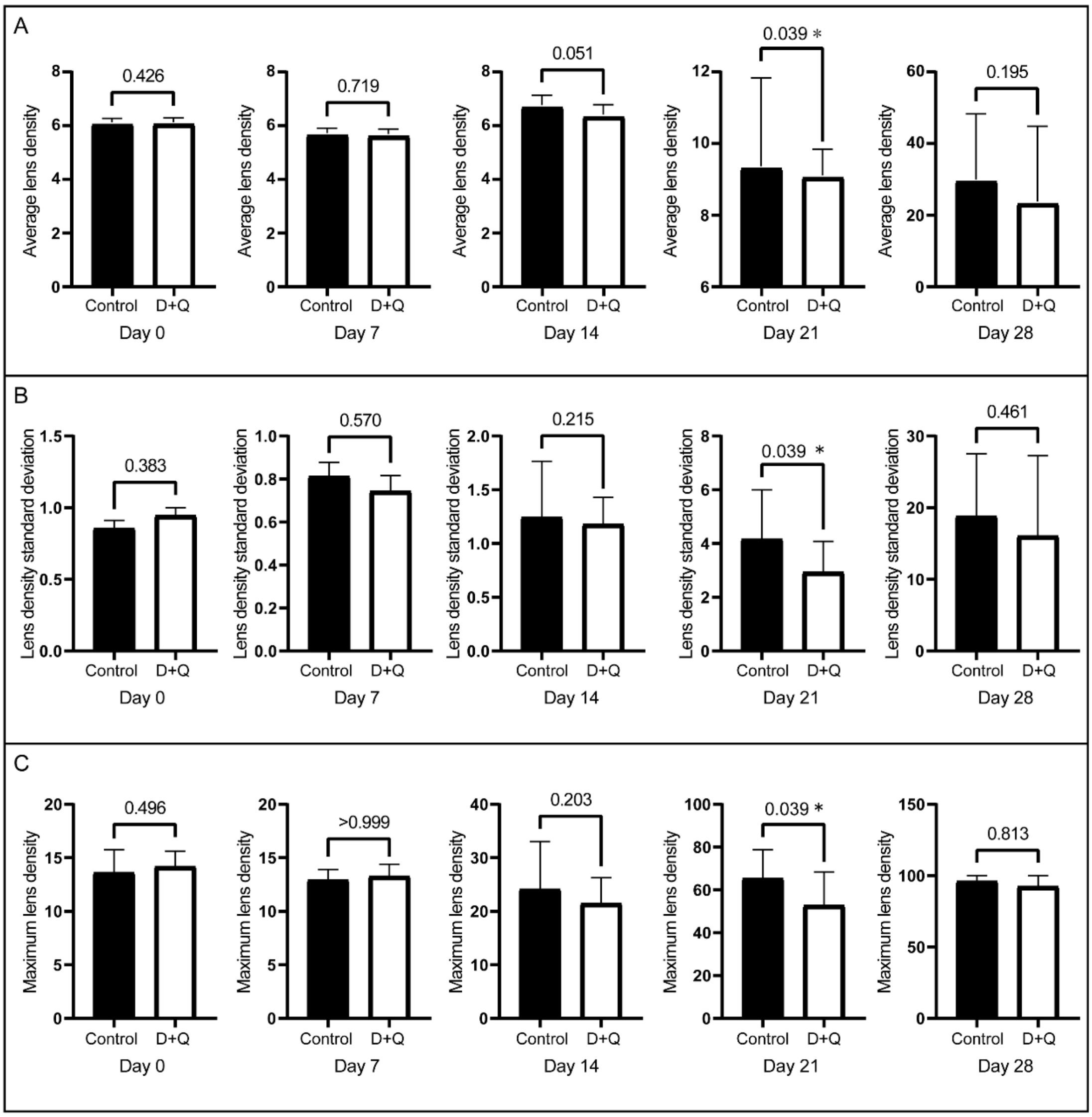

Figure S5. The comparisons between paired eyes from each cataract rats after administration of D+Q with high concentration. A, the comparison of average lens density between paired eyes. B, the comparison of lens density standard deviation between paired eyes. C, the comparison of maximum lens density between paired eyes. D, dasatinib; Q, quercetin.

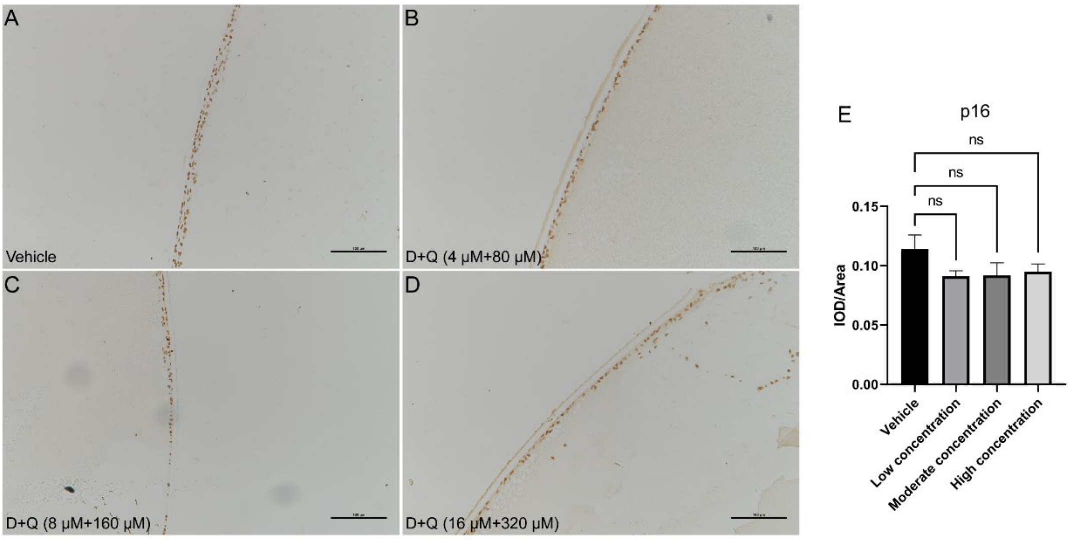

Figure S6. The expression of p16 in cataract LECs reduced after administration of D+Q.

A-D, the IHC staining of p16 in cataract lenses after administration of D+Q and vehicle.

E, the expression level of p16 in cataract LECs after administration of D+Q and vehicle.

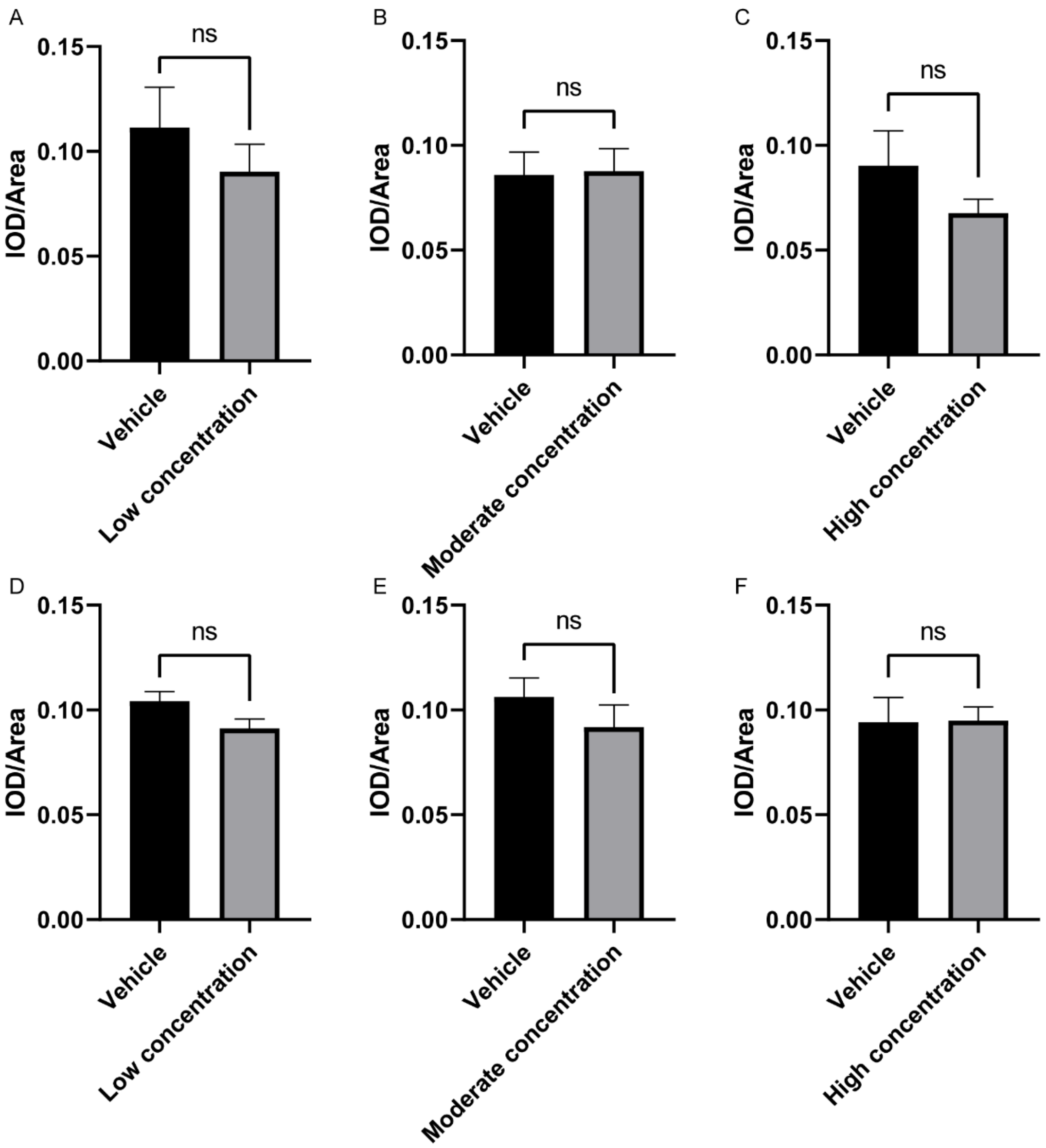

Figure S7. The expression level of γH2AX and p16 between paired eyes from each cataract rats after administration of D+Q. A-C, the expression level of γH2AX in different concentration of D+Q. D-F, the expression level of p16 in different concentration of D+Q.

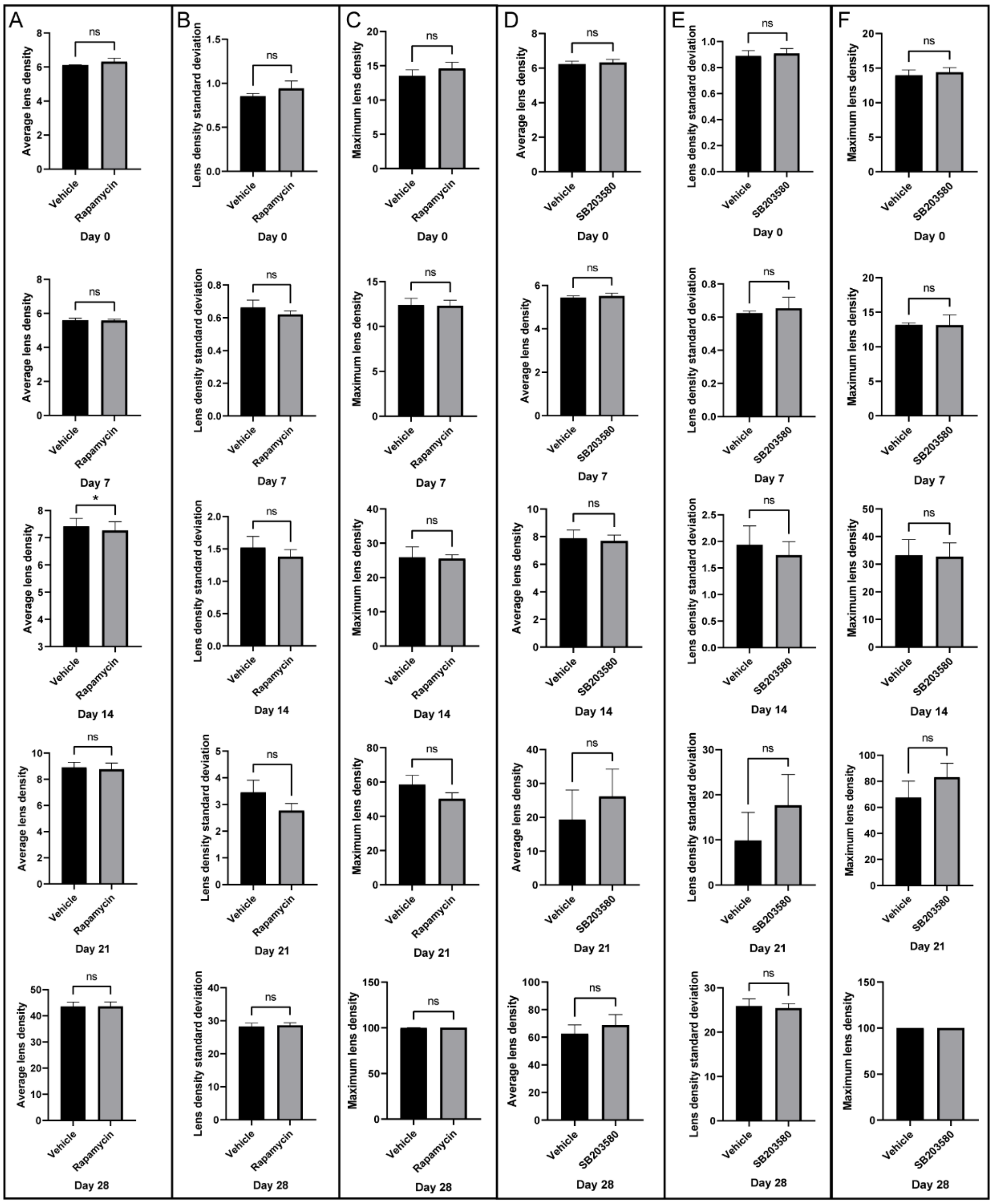

Figure S8. The comparisons between paired eyes from each cataract rats after administration of SASP inhibitors. A-C, the comparison of ALD, LDSD, and MLD between paired eyes after administration of rapamycin. D-F, the comparison of ALD, LDSD, and MLD between paired eyes after administration of SB203580. ALD, average lens density; LDSD, lens density standard deviation; MLD, maximum lens density
